# Large-scale countrywide screening for tick-borne pathogens in field-collected ticks in Latvia during 2017–2019

**DOI:** 10.1186/s13071-020-04219-7

**Published:** 2020-07-14

**Authors:** Valentina Capligina, Maija Seleznova, Sarmite Akopjana, Lauma Freimane, Marija Lazovska, Rudolfs Krumins, Agnija Kivrane, Agne Namina, Darja Aleinikova, Janis Kimsis, Alisa Kazarina, Viktorija Igumnova, Antra Bormane, Renate Ranka

**Affiliations:** Latvian Biomedical Research and Study Centre, Ratsupites Street 1, Riga, Latvia

**Keywords:** *Ixodes ricinus*, *Ixodes persulcatus*, *Dermacentor reticulatus*, Tick-borne pathogens, Latvia

## Abstract

**Background:**

Tick-borne diseases are of substantial concern worldwide in both humans and animals. Several hard tick species are of medical and veterinary interest in Europe, and changes in the range of tick species can affect the spread of zoonotic pathogens. The aim of the present study was to map the current prevalence and distribution pattern of ticks and related tick-borne pathogens in Latvia, a Baltic state in northern Europe.

**Methods:**

Nearly 4600 *Ixodes ricinus*, *I. persulcatus* and *Dermacentor reticulatus* tick samples were collected in all regions of Latvia during 2017–2019 and were screened by molecular methods to reveal the prevalence and distribution pattern of a wide spectrum of tick-borne pathogens.

**Results:**

New localities of *D. reticulatus* occurrence were found in western and central Latvia, including the Riga region, indicating that the northern border of *D. reticulatus* in Europe has moved farther to the north. Among the analyzed ticks, 33.42% carried at least one tick-borne pathogen, and 5.55% of tick samples were positive for two or three pathogens. A higher overall prevalence of tick-borne pathogens was observed in *I. ricinus* (34.92%) and *I. persulcatus* (31.65%) than in *D. reticulatus* (24.2%). The molecular analysis revealed the presence of tick-borne encephalitis virus, *Babesia* spp., *Borrelia* spp., *Anaplasma phagocytophilum* and *Rickettsia* spp. Overall, 15 and 7 tick-borne pathogen species were detected in *Ixodes* spp. and *D. reticulatus* ticks, respectively. This is the first report of *Borrelia miyamotoi* in Latvian field-collected ticks.

**Conclusions:**

This large-scale countrywide study provides a snapshot of the current distribution patterns of *Ixodes* and *Dermacentor* ticks in Latvia and gives us a reliable overview of tick-borne pathogens in Latvian field-collected ticks.
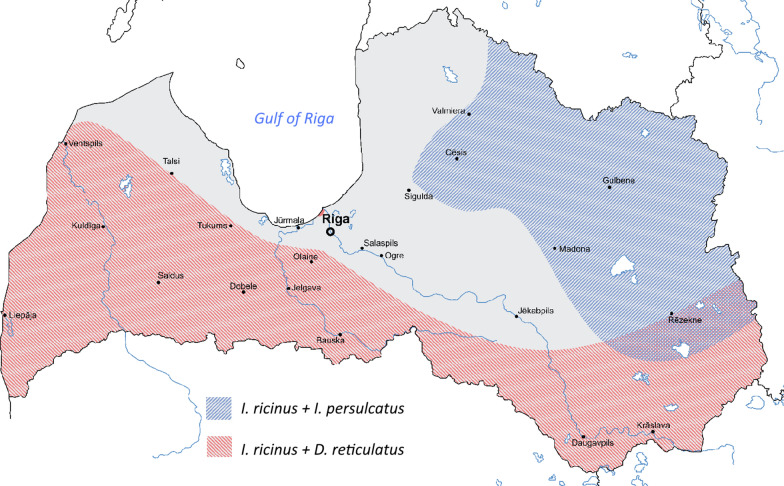

## Background

Ticks are blood-feeding ectoparasites that are found worldwide. There is evidence that ticks parasitized dinosaurs almost 99 million years ago [1]. Ticks are the primary vectors of many viral and bacterial pathogens, protozoans and helminths, which pose significant threats to human and animal health [2]. Different tick species are able to transmit different diseases, and in-depth cellular and molecular studies of both tick physiology and the pathogens that they transmit show that several intrinsic factors play an important role in the maintenance of the pathogens and the vector competence of tick species [3, 4]. According to data from the European Centre for Disease Prevention and Control (ECDC), there are three hard tick species of medical and veterinary interest in north-eastern Europe: *Ixodes ricinus*; *I. persulcatus*; and *Dermacentor reticulatus* [5]. These ticks are carriers of a number of epidemiologically significant pathogens, such as *Anaplasma phagocytophilum*, *Babesia* spp., *Borrelia* spp., tick-borne encephalitis virus (TBEV), *Rickettsia* spp., *Francisella tularensis*, *Bartonella* spp., and *Coxiella burnetii* [6–9].

In Latvia, a Baltic state in northern Europe, three tick-borne diseases are registered annually: Lyme disease (borreliosis, 481 cases in 2018, 24.9 cases per 100,000 individuals), tick-borne encephalitis (TBE; 169 cases in 2018, 8.7 cases per 100,000 individuals), and granulocytic ehrlichiosis, or anaplasmosis (27 cases in 2018, 1.4 cases per 100,000 individuals) (data from the Centre for Disease Prevention and Control of Latvia; https://spkc.gov.lv/). Babesiosis is not registered in Latvia as a human disease; however, it is playing an increasingly important role in veterinary medicine [10]. During the last decade, in addition to the commonly recorded *I. ricinus* and *I. persulcatus* ticks, the appearance and spread of *D. reticulatus* populations was reported in Latvia [11]. However, the current tick distribution and the diversity of tick-associated pathogens have not been fully characterized. Here, we present the results of large-scale, countrywide screening for tick-borne pathogens in field-collected ticks in Latvia during 2017–2019.

## Methods

### Tick collection

Host-seeking ticks were collected from ground vegetation by flagging during the tick activity period during 2017–2019 in all regions of Latvia. Tick sampling was conducted in different habitats, such as open landscapes, forests, abandoned fields and ecotones. The collection sites were geolocated. Maps were created using the publicly available Google Earth platform (http://www.google.co.uk/intl/en_uk/earth).

Tick samples were preserved in 70% ethanol. Ticks were identified to the species level, and their stage and sex were identified based on morphological characteristics [12, 13]. After morphological identification, the ticks were individually stored at − 20 °C.

### DNA and RNA extraction

For DNA/RNA extraction, all samples were processed individually. The ticks were washed with 70% ethanol, dried, transferred to individual tubes and crushed in 300 μl of sterile water. For DNA isolation, 50 μl of digestion buffer [30 mM Tris-HCl (pH 8.0), 75 mM EDTA (pH 8.0), 0.3 M NaCl, 1.5% SDS] and 2.5 μl of proteinase K (20 mg/ml) were added to 100 μl of a tick suspension, and the mixture was incubated at 50 °C for 1 h. DNA was extracted *via* the phenol/chloroform method and stored at -20 °C. Total RNA was extracted from a 100 μl aliquot of a tick suspension using QIAzol reagent (Qiagen, Hilden, Germany), as described by the manufacturer. The RNA pellet was dissolved in 50 μl of nuclease-free water (Fermentas, Vilnius, Lithuania) and stored at − 70 °C.

### PCR assays

All samples were tested for the presence of *Babesia* spp., *Borrelia* spp., *A. phagocytophilum* and *Rickettsia* spp. using a nested polymerase chain reaction (PCR) targeting the *18S* rRNA, *16S* rRNA and *gltA* genes, respectively, as previously described [10]. The presence of TBEV RNA in the samples was detected by reverse transcription real-time polymerase chain reaction (RT-qPCR) amplifying a fragment of the 3’ NCR (non-coding region) as previously described [14].

To prevent PCR amplicon contamination, sample DNA/RNA preparation, reaction preparation, PCR amplification, and amplicon detection were all performed in separate areas using filter tips. Both negative and positive controls were included in all PCR amplification steps. As a negative control, PCR mixtures without DNA were used. As positive controls for PCR, the following specimens were used: *Borrelia burgdorferi* (*sensu stricto*) B31 strain (kindly donated by S. Bergström, Umeå, Sweden); *A. phagocytophilum* Webster strain (kindly donated by Friederike von Loewenich, Institute of Medical Microbiology, University of Freiburg, Germany); *Rickettsia helvetica*-positive tick sample Lv-P44, which we acquired in a previous study [15]; and *Babesia canis*-positive clinical sample Lv-dog 2 (positive DNA sample from dog blood), which we also detected in a previous study [16]. All primers and probes were synthesized by Metabion International AG (Munich, Germany) and all PCR reagents were purchased from Thermo Fisher Scientific (Waltham, MA, USA). The PCR products were visualized by electrophoresis in 1.5% agarose gels (TopVision Agarose; Thermo Fisher Scientific) in 1× Tris-acetate-EDTA buffer containing 0.2 μg/ml ethidium bromide under UV light.

### Sequencing

Positive PCR amplicons were purified and analyzed by Sanger sequencing from both DNA strands using the BigDye Terminator v3.1 Cycle Sequencing Kit (Applied Biosystems, Waltham, USA) in an ABI Prism 3100 Genetic Analyzer (Perkin- Elmer, Waltham, USA). Sequence chromatograms were viewed and edited using Finch TV Version 1.4.0 software (Geospiza Inc., Seattle, USA). Primer sequences were omitted in all sample sequences. Pathogens were identified using the NCBI GenBank database (http://www.ncbi.nlm.nih.gov/genbank) and BLAST (Basic Local Alignment Search Tool) software [17] (http://www.ncbi.nlm.nih.gov/BLAST). Pathogen genotypes were assigned based on sequence similarity (99–100%) with the corresponding genes of the reference strains.

### Statistical analysis

All ticks were processed individually, and their prevalence was expressed as a percentage. The prevalence of pathogens was calculated with the 95% confidence intervals of a proportion by the “exact” method of Clopper and Pearson (GraphPad Prism 6; GraphPad Software, La Jolla, CA, USA). A *P*-value was calculated using the two-sided Fisher’s exact test (GraphPad Prism 6). *P*-values were adjusted for multiple testing by Holm correction in R using the R statistical package [18]. Values of *P* ≤ 0.05 were considered significant.

## Results

### Distribution of tick species

**Fig. 1:**
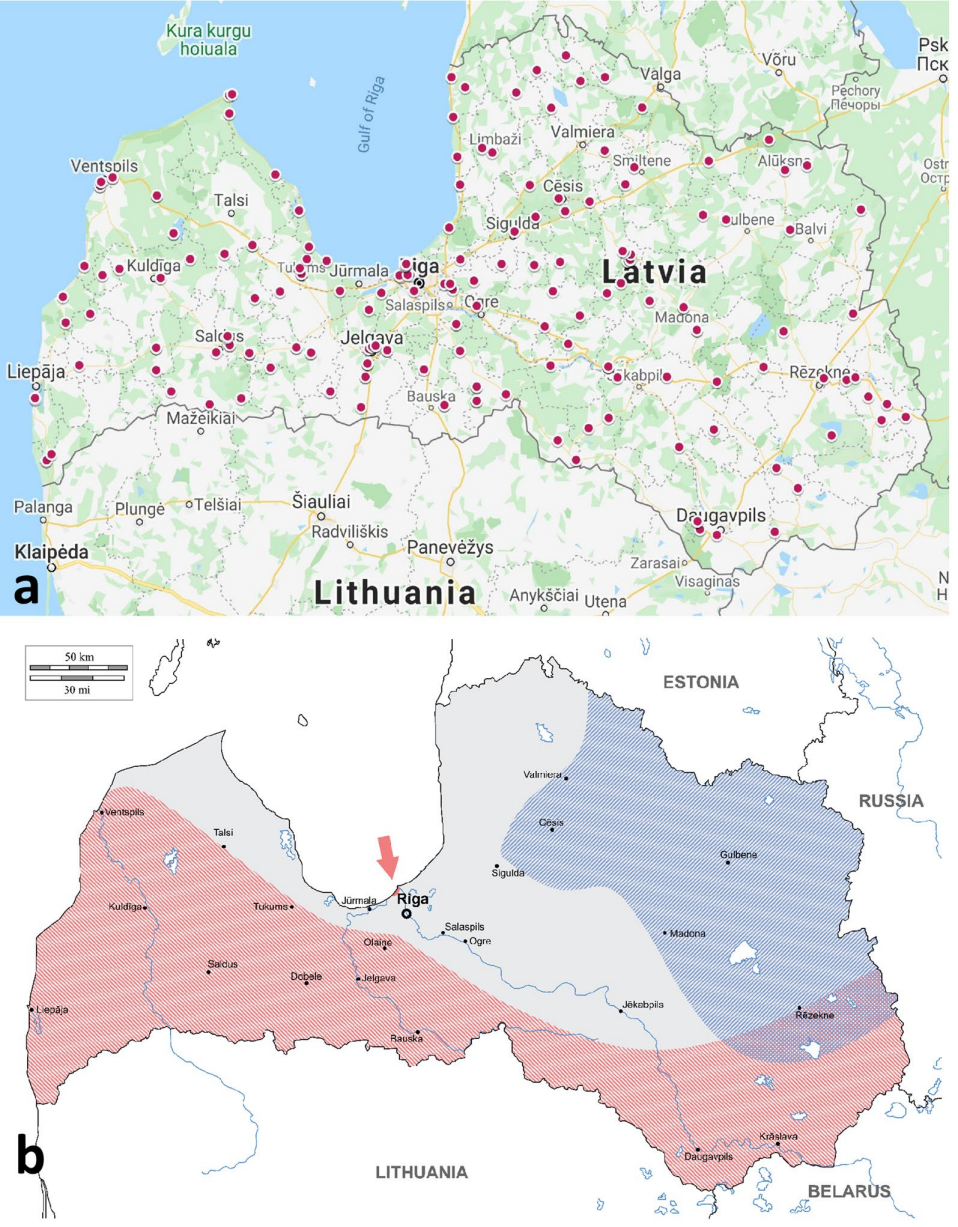
Tick collection sites and distribution of tick species in Latvia. **a** Map of Latvia with the 189 tick sample sites, 2017–2019. Data were mapped using Google Earth. **b** Distribution of tick species in Latvia. The sympatric area for *Ixodes persulcatus* and *I. ricinus* tick species is highlighted in blue. The sympatric area for *Dermacentor reticulatus* and *I. ricinus* tick species is highlighted in red

In total, 4593 ticks were collected from 189 locations in Latvia (Fig. 1a). These ticks belonged to three species: *I. ricinus* (3840 samples: 1363 males, 1324 females and 1153 nymphs), *I. persulcatus* (158 samples: 77 males, 74 females and 7 nymphs), and *D. reticulatus* (595 samples: 172 males, 419 females and 4 nymphs). Based on geolocation data, the distribution of these species in Latvia was mapped. As expected, *I. ricinus* was present across the whole country, and the distribution of *I. persulcatus* was restricted to the eastern and northern-eastern Vidzeme and Latgale regions (Fig. 1b). *Dermacentor reticulatus* ticks were collected in the southern, central and western regions of Latvia. In addition, *D. reticulatus* was detected in geographically separate small localities in the Riga region (Fig. 1b, arrow). These results indicated that sympatric populations of *D. reticulatus* and *I. ricinus* ticks as well as *D. reticulatus*, *I. ricinus* and *I. persulcatus* ticks exist in several regions of Latvia. Additionally, a shift northward in the distribution of *D. reticulatus* in Latvia was observed.

### Pathogen prevalence in tick species

**Fig. 2:**
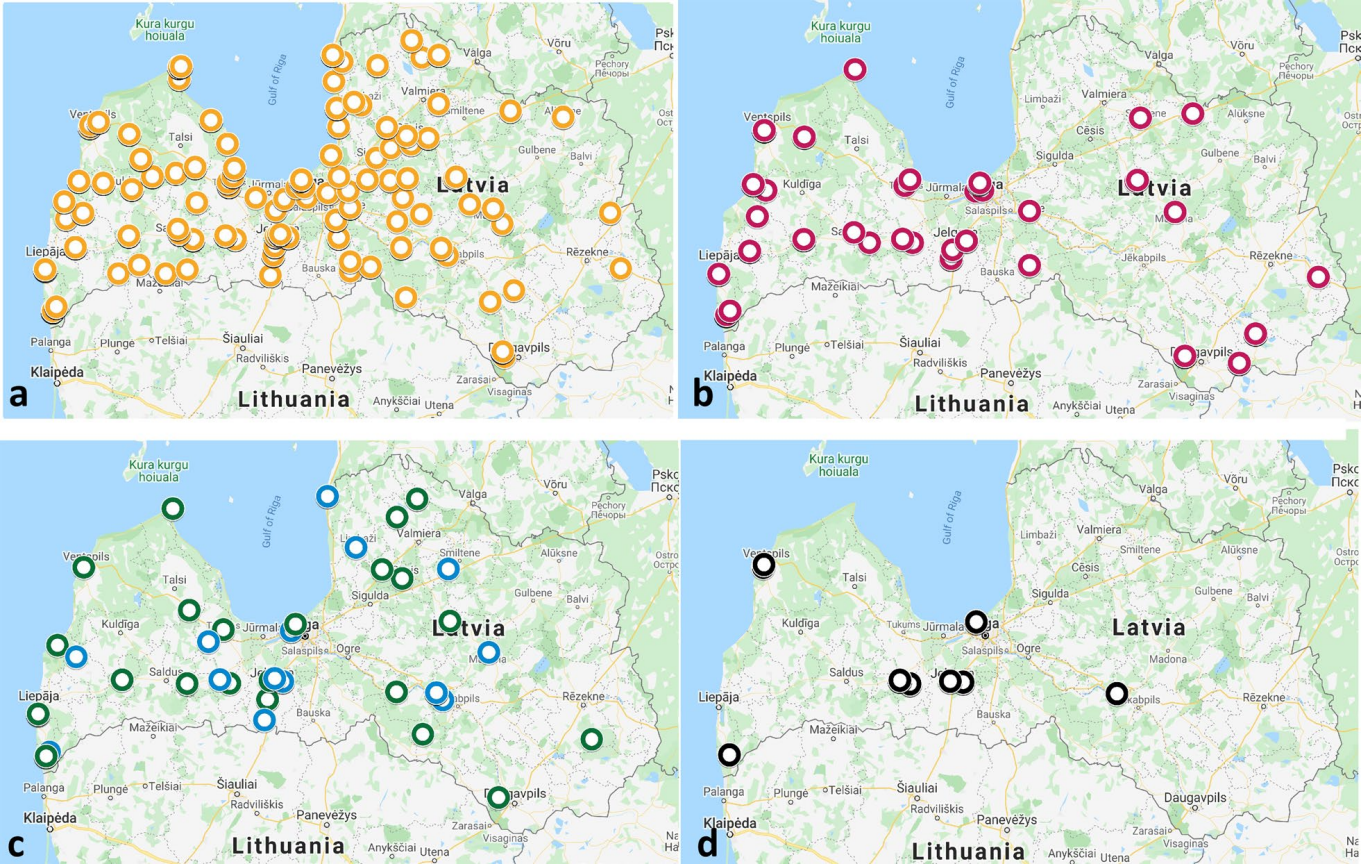
Distribution of *Rickettsia* spp. and *Babesia* spp. in Latvia. **a***Rickettsia helvetica* (orange circles). **b***Rickettsia raoultii* (red circles). **c***Babesia microti* (blue circles) and *Babesia venatorum* (green circles). **d***Babesia canis* (black circles)

**Fig. 3:**
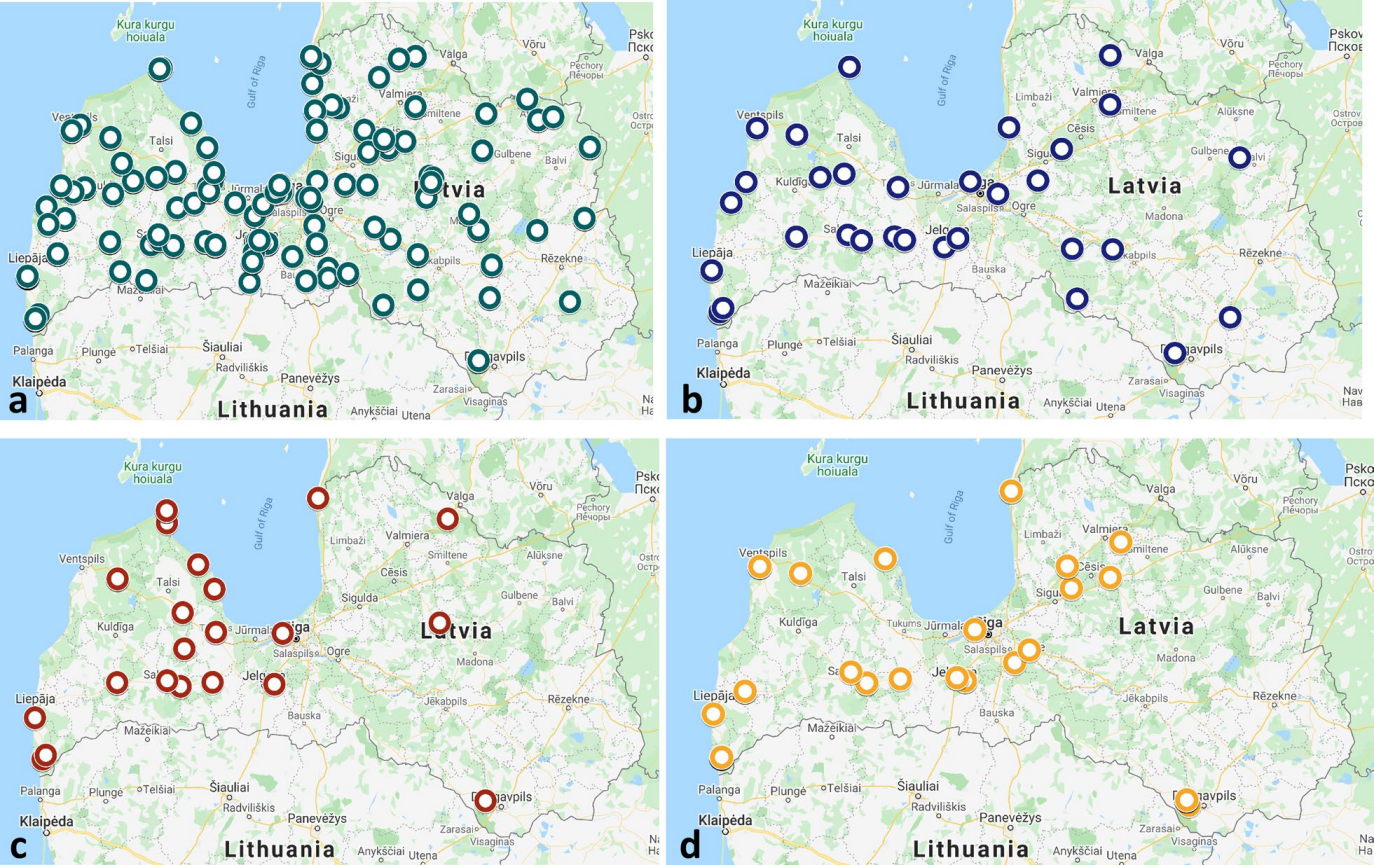
Distribution of *Borrelia* spp., *Anaplasma phagocytophilum* and tick-borne encephalitis virus in Latvia. **a***Borrelia burgdorferi* (*s.l.*) (green circles). **b***Borrelia miyamotoi* (blue circles). **c** Tick-borne encephalitis virus (TBEV) (red circles). **d***Anaplasma phagocytophilum* (yellow circles)

The prevalence of tick-borne pathogens was studied and compared in the different tick species. The results are presented in Table 1. The distribution of different pathogens is presented in Figs. 2 and 3. In total, 33.42% (1535/4593) of the field-collected ticks were pathogen-positive (34.92% of *I. ricinus*; 31.65% of *I. persulcatus*; and 24.2% of *D. reticulatus*) and the differences were statistically significant (OR: 1.671, 95% CI: 1.37–2.038, *P* < 0.0001) (Table 1). As expected, nymphs were pathogen-positive less often, and the pathogen load did not differ between males and females. Moreover, no pathogens were detected in *D. reticulatus* nymphs in our study.

**Table 1:** Prevalence of pathogens in field-collected ticks in Latvia, 2017–2019 (*n* = 4593)

Pathogen species^a^	Tick species													*P-*value^b^
	*Ixodes ricinus* (95% CI)				*Ixodes persulcatus* (95% CI)				*Dermacentor reticulatus* (95% CI)				All ticks	
	Male	Female	Nymph	Total	Male	Female	Nymph	Total	Male	Female	Nymph	Total		
TBEV	0.88	0.6	0.43	0.65 (0.44–0.96)	1.3	0	0	0.63 (0.01–3.86)	1.74	0.24	0	0.67 (0.2–1.78)	0.65	1.000
*Babesia* total*	3.08	3.25	1.04	2.53 (2.07–3.07)	0	1.35	0	0.63 (0.01–3.86)	0	0.48	0	0.34 (0.01–1.30)	2.18	0.0002*
*Ba. canis*	1.39	0.76	0.52	0.91 (0.65–1.27)	0	0	0	0 (0–2.86)	0	0.48	0	0.34 (0.01–1.30)	0.81	0.2727
*Ba. microti*	0.29	1.28	0	0.55 (0.35–0.84)	0	1.35	0	0.63 (0.01–3.86)	0	0	0	0 (0–0.77)	0.48	0.1081
Ba. venatorum*	1.39	1.21	0.52	1.07 (0.78–1.45)	0	0	0	0 (0–2.86)	0	0	0	0 (0–0.77)	0.89	0.0075*
*Ba. capreoli*	0	0.08	0	0.03 (0.01–0.16)	0	0	0	0 (0–2.86)	0	0	0	0 (0–0.77)	0.02	1.000
*A. phagocytophilum*	1.39	1.44	0.69	1.20 (0.90–1.60)	1.3	0	0	0.63 (0.01–3.86)	0.58	0.48	0	0.50 (0.10–1.54)	1.09	0.3050
*Rickettsia* total*	20.25	21.22	16.48	19.45 (18.23–20.74)	7.79	5.41	28.57	7.59 (4.28–12.92)	26.16	21.48	0	22.69 (19.50–26.23)	19.46	< 0.0001*
R. helvetica*	18.86	18.73	15.35	17.76 (16.58–19.00)	6.49	5.41	14.29	6.33 (3.34–11.39)	8.14	4.06	0	5.21 (3.67–7.32)	15.74	< 0.0001*
*R. monacensis*	0.07	0.15	0.17	0.13 (0.05–0.31)	0	0	0	0 (0–2.86)	0	0	0	0 (0–0.77)	0.11	1.000
R. raoultii*	1.98	2.79	1.47	2.11 (1.70–2.62)	1.3	0	14.29	1.27 (0.05–4.79)	18.6	17.66	0	17.82 (14.94–21.10)	4.11	< 0.0001*
Bo. miyamotoi*	1.39	1.51	0.43	1.15 (0.85–1.54)	0	2.70	0	1.27 (0.05–4.79)	0	0	0	0 (0–0.77)	1.0	0.0062*
*Bo. burgdorferi* (*s.l.*)*	20.47	21.15	4.77	15.99 (14.86–17.18)	20.78	27.03	14.29	23.42 (17.46–30.63)	0	1.43	0	1.01 (0.41–2.24)	14.3	< 0.0001*
Bo. afzelii*	11.96	12.08	3.12	9.35 (8.47–10.31)	12.99	17.57	14.29	15.19 (10.37–21.67)	0	1.19	0	0.84 (0.3–2.01)	8.45	< 0.0001*
*Bo. burgdorferi* (*s.s.*)	0.66	0.83	0.26	0.60 (0.39–0.90)	0	0	0	0 (0–2.86)	0	0	0	0 (0–0.77)	0.5	0.1367
Bo. garinii*	2.86	3.55	1.13	2.58 (2.12–3.13)	7.79	10.81	0	8.86 (5.25–14.43)	0	0.24	0	0.17 (0–0.01)	2.48	< 0.0001*
Bo. lusitaniae*	3.89	3.02	0.26	2.50 (2.05–3.05)	0	0	0	0 (0–2.86)	0	0	0	0 (0–0.77)	2.09	< 0.0001*
Bo. valaisiana*	1.39	2.04	0.09	1.22 (0.92–1.63)	0	0	0	0 (0–2.86)	0	0	0	0 (0–0.77)	1.02	0.00285*
Any pathogen-positive*	40.13	40.63	22.2	34.92 (33.43–36.44)	29.87	33.78	28.57	31.65 (24.89–39.27)	27.33	23.15	0	24.2 (20.93–27.81)	33.42	< 0.0001*
Total no. of ticks	1363	1324	1153	3840	77	74	7	158	172	419	4	595	4593	

^a^Including co-infections

^b^*P*-value was calculated for the total numbers

*Statistically significant difference (*P* ≤ 0.05)

*Abbreviations*: CI, confidence interval; TBEV, tick-borne encephalitis virus

The molecular analysis indicated the presence of 15 tick-borne pathogens in *Ixodes* ticks: TBEV; *Ba. canis*; *Ba. microti*; *Ba. venatorum*; *Ba. capreoli*; *A. phagocytophilum*; *R. helvetica*; *R. monacensis*; *R. raoultii*; *Bo. miyamotoi*; *Bo. afzelii*; *Bo. burgdorferi* (*s.s.*); *Bo. garinii*; *Bo. lusitaniae*; and *Bo. valaisiana*. In *D. reticulatus* ticks, 7 pathogens were detected: TBEV; *Ba. canis*; *R. helvetica*; *R. raoultii*; *Bo. afzelii*; *Bo. garinii*; and *A. phagocytophilum*.

### Prevalence of *Rickettsia* spp.

*Rickettsia* spp. were detected in the tick samples most frequently: 19.46% (894/4593) of the ticks were *Rickettsia-*positive. *Ixodes persulcatus* ticks were *Rickettsia-*positive significantly less frequently (7.59%) than *I. ricinus* (19.45%) and *D. reticulatus* (22.69%) (OR: 3.02, 95% CI: 1.669–5.466, *P* < 0.0001) (Table 1). Among *Rickettsia* species, *R. helvetica* was most frequently detected; in total, 15.74% of ticks were positive for this species, and *R. helvetica*-positive samples were found in all regions of Latvia (Fig. 2a). The prevalence of *R. helvetica* in *I. ricinus* ticks was significantly greater (17.76%) than that in either *I. persulcatus* (6.33%) or *D. reticulatus* (5.21%) ticks (OR: 3.75, 95% CI: 2.708–5.193, *P* < 0.0001). On the other hand, *R. raoultii* was associated mainly with *D. reticulatus*, in which this pathogen was detected in 17.82% of samples, in comparison with 2.11% of *I. ricinus* and 1.27% of *I. persulcatus* ticks (OR: 0.098, 95% CI: 0.072–0.132, *P* < 0.0001). *Rickettsia monacensis* was detected only in 5 *I. ricinus* samples (0.13%) (Table 1).

### Prevalence of *Babesia* spp.

The prevalence of *Babesia* pathogens was significantly higher in *I. ricinus* ticks than in *I. persulcatus* and *D. reticulatus* (2.53% *vs* 0.63% and 0.34%) (OR: 0.154, 95% CI: 0.049–0.488, *P* = 0.0002) (Table 1). In total, four *Babesia* species were identified in *I. ricinus* (*Ba. canis*, *Ba. microti*, *Ba. venatorum* and *Ba. capreoli*). *Babesia microti* and *Ba. canis* were the only *Babesia* species detected in *I. persulcatus* and *D. reticulatus*, respectively. Surprisingly, very similar proportions of field-collected *I. ricinus* and *D. reticulatus* ticks were *Ba. canis-*positive in this study (0.91% *vs* 0.34%) (OR: 2.7269, 95% CI: 0.6974–23.4610, *P* = 0.2223). However, *Ba. canis*-positive samples were found only in areas with the sympatric occurrence of *I. ricinus* and *D. reticulatus* (Fig. 1d). Additionally, a non-uniform distribution pattern was observed for *Ba. canis*, as *Ba. canis*-positive samples tended to form geographically separate, condensed foci independent of the tick species (Fig. 1d).

### Prevalence of TBEV

The three tick-borne pathogens that cause the most common tick-borne diseases in Latvia, i.e. TBEV, *Bo. burgdorferi* (*s.l.*) and *A. phagocytophilum*, were all detected in tick samples in our study. Importantly, TBEV was detected in nearly identical proportions of *I. ricinus*, *I. persulcatus* and *D. reticulatus* samples (0.65, 0.63 and 0.67%, respectively) (Table 1). The geolocation data revealed that TBEV-positive ticks were found mostly in the western part of Latvia (Kurzeme region), and the TBEV distribution was markedly lower in northern and eastern regions (Fig. 3c).

### Prevalence of *A. phagocytophilum*

The etiological agent of anaplasmosis, *A. phagocytophilum*, was found in 1.2, 0.63 and 0.5% of *I. ricinus*, *I. persulcatus* and *D. reticulatus* ticks, respectively; the differences were not statistically significant. This pathogen was detected mainly in ticks in the western, central and northern regions of Latvia (Fig. 3d).

### Prevalence of *Bo. burgdorferi* (*s.l.*)

The geolocation data showed that Lyme disease-causing pathogens were present in host-seeking ticks throughout the territory of Latvia (Fig. 3a). *Borrelia burgdorferi* (*s.l.*) group spirochaetes were detected in 15.99% and 23.42% of *I. ricinus* and *I. persulcatus* ticks, respectively, while only 1.01% (6/595) of *D. reticulatus* were *Borrelia*-positive in this study; this difference was statistically significant (OR: 19.094, 95% CI: 8.506–42.86, *P* < 0.0001) (Table 1). Also, *I. persulcatus* ticks were *Bo. burgdorferi* (*s.l.*) positive significantly more frequently than *I. ricinus* (OR: 0.6225, 95% CI: 0.4228–0.9352, *P* = 0.0203). Within this pathogen group, the genotyping analysis revealed the presence of *Bo. afzelii*, *Bo. burgdorferi* (*s.s.*), *Bo. garinii*, *Bo. lusitaniae* and *Bo. valaisiana*. The most abundant species was *Bo. afzelii*, which was detected in 9.35 and 15.19% of *I. ricinus* and *I. persulcatus* ticks, respectively. *Borrelia afzelii* was also the only Lyme disease-causing spirochaete present in several *D. reticulatus* ticks (5/595, 0.84%) (Table 1). Additionally, 2.58% and 8.86% of *I. ricinus* and *I. persulcatus* were *Bo. garinii*-positive, while *Bo. lusitaniae*, *Bo. valaisiana* and *Bo. burgdorferi* (*s.s.*) were detected in 2.5%, 1.22% and 0.6% of *I. ricinus* ticks.

### Prevalence of *Bo. miyamotoi*

The hard-tick-borne relapsing fever-causing pathogen *Bo. miyamotoi* was present solely in *Ixodes* ticks (1.15% and 1.27% of *I. ricinus* and *I. persulcatus*, respectively); no *Bo. miyamotoi*-positive *D. reticulatus* samples were found in this study (Table 1). Similar to the Lyme disease spirochaetes, *Bo. miyamotoi* was present in all regions of Latvia (Fig. 3b).

### Prevalence of co-infections in ticks

In our study, 5.55% (255/4593) of the host-seeking tick samples were positive for more than one tick-borne infectious agent. Co-infection with two or three tick-borne pathogens, including two genotypes of the *Bo. burgdorferi* (*s.l.*) group, was detected in 6.3%, 3.16% and 1.34% of the *I. ricinus*, *I. persulcatus* and *D. reticulatus* samples, respectively, and the difference between *I. ricinus* and *D. reticulatus* was statistically significant (OR: 4.9341, 95% CI: 2.4462–11.6218, *P* < 0.0001) (Table 2). In total, 57 different pathogen combinations were detected (Additional file 1: Table S1). The most common co-infection detected in tick samples involved *Bo. burgdorferi* (*s.l.*) and *Rickettsia* spp. (2.94%). Importantly, co-infection of *Bo. burgdorferi* (*s.l.*) with TBEV was found in 0.1% and 0.63% of *I. ricinus* and *I. persulcatus* ticks, and co-infection with three pathogens was detected in 0.55% (21/3840) of *I. ricinus* ticks in our study (Table 2).

**Table 2:** Prevalence of co-infections in field-collected ticks in Latvia, 2017–2019 (*n* = 4593)

Pathogen co-infections	*I. ricinus* (95% CI)				*I. persulcatus* (95% CI)				*D. reticulatus* (95% CI)				All ticks	*P*-value ^a^
	Male	Female	Nymph	Total	Male	Female	Nymph	Total	Male	Female	Nymph	Total		
*Bo. burgdorferi* (*s.l*.) + Rickettsia*	3.96	5.14	0.78	3.41 (2.88–4.04)	0	1.35	14.29	1.27 (0.05–4.79)	0	0.48	0	0.34 (0.01–1.30)	2.94	< 0.0001*
*Bo. burgdorferi* (*s.l*.) + *Babesia*	0.88	0.91	0.09	0.65 (0.44–0.96)	0	1.35	0	0.63 (0.01–3.86)	0	0	0	0 (0–0.77)	0.57	0.0883
*Rickettsia* two species	0.59	0.30	0.43	0.44 (0.27–0.71)	0	0	0	0 (0–2.86)	0.58	0.24	0	0.34 (0.01–1.30)	0.41	1.0000
*Rickettsia* + *A. phagocytophilum*	0.51	0.15	0.09	0.26 (0.13–0.49)	0	0	0	0 (0–2.86)	0	0	0	0 (0–0.77)	0.22	0.5609
*Rickettsia* + *Babesia*	0.44	0.23	0	0.23 (0.12–0.45)	0	0	0	0 (0–2.86)	0	0.48	0	0.34 (0.01–1.30)	0.22	0.7622
*Bo. burgdorferi* (*s.l*.) two species	0.22	0.30	0	0.18 (0.08–0.38)	0	1.35	0	0.63 (0.01–3.86)	0	0	0	0 (0–0.77)	0.17	0.2195
*Bo. miyamotoi* + *Rickettsia*	0.22	0.23	0.09	0.18 (0.08–0.38)	0	0	0	0 (0–2.86)	0	0	0	0 (0–0.77)	0.15	0.6901
TBEV + *Rickettsia*	0.15	0.08	0.09	0.10 (0.03–0.28)	0	0	0	0 (0–2.86)	1.16	0	0	0.34 (0.01–1.30)	0.13	0.3410
TBEV + *Bo. burgdorferi* (*s.l*.)	0.22	0.08	0	0.10 (0.03–0.28)	1.30	0	0	0.63 (0.01–3.86)	0	0	0	0 (0–0.77)	0.11	0.1770
Two pathogens others	0.22	0.30	0	0.18 (0.08–0.38)	0	0	0	0 (0–2.86)	0	0	0	0 (0–0.77)	0.15	0.6901
Two pathogens total*	7.41	7.70	1.56	5.76 (5.06–6.54)	1.30	4.05	14.29	3.16 (1.16–7.39)	1.74	1.19	0	1.34 (0.63–2.68)	5.09	< 0.0001*
Three pathogens total	0.44	0.83	0.35	0.55 (0.35–0.84)	0	0	0	0 (0–2.86)	0	0	0	0 (0–0.77)	0.46	0.1565
Co-infections total*	7.85	8.53	1.91	6.30 (5.58–7.12)	1.30	4.05	14.29	3.16 (1.16–7.39)	1.74	1.19	0	1.34 (0.63–2.68)	5.55	< 0.0001*
Total no. of ticks	1363	1324	1153	3840	77	74	7	158	172	419	4	595	4593	

^a^*P*-value was calculated for the total numbers

*Statistically significant difference (*P* ≤ 0.05)

*Abbreviations*: CI, confidence interval; TBEV, tick-borne encephalitis virus

## Discussion

Here, the distribution of three tick species, *I. ricinus*, *I. persulcatus* and *D. reticulatus*, and the prevalence of tick-associated pathogens in Latvia was investigated. This is the most comprehensive countrywide study including host-seeking tick samples collected during 2017–2019.

### Distribution of tick species in Latvia

As expected, all three epidemiologically important tick species were present in Latvia. The distribution of *I. persulcatus* was in accord with previous observations, as this tick species was found in northern and eastern parts of the country. The geolocation data of the tick collection sites revealed new localities of *D. reticulatus* occurrence in western and central Latvia, in addition to the previously observed locations [11]. Thus, the present study demonstrates that during the past few years, *D. reticulatus* has efficiently expanded its range in Latvia, and the northern border of *D. reticulatus* in Europe has moved farther north. Moreover, *D. reticulatus* has been detected in geographically separate new localities in the Riga region, indicating the development of new foci outside of the major distribution area. Similar to these findings, we reported in a recent study that from 2011 to 2016, *D. reticulatus* ticks were removed from dogs in western, southern and central parts of Latvia and the Riga region [10]. Outside of Latvia, the spread of *D. reticulatus* during the last decade has been notable in other European countries such as neighboring Lithuania [9, 11, 19]. Ticks spend most of their life in the environment, and all tick stages are dependent on a combination of climate variables, vegetation and the distribution and availability of suitable hosts. Climate change associated with warmer temperatures has a positive effect on tick survival in nature during non-parasitic periods of the life-cycle and can contribute to an increase in tick populations and the probability of spreading, establishment and survival in new geographical areas [4, 20].

Increases in human travel, animal transport, and environmental changes, including the establishment of new tick foci, are responsible for the emergence and/or spread of numerous tick-borne pathogens [21]. In Europe, ticks are the most important vectors of human and animal infectious diseases and transmit more pathogens than any other arthropod [22]. Effective tick-based surveillance is essential for monitoring human and/or animal disease emergence [23]. However, studies on the prevalence of tick-borne pathogens in ticks are not available for all European countries, and the current tick situation in Latvia has not been fully characterized. Previous investigations of field-collected ticks in Latvia have been limited to *Ixodes* ticks and only a few pathogens and/or geographical locations, and the most recent studies on the prevalence of the highly medically important *Borrelia* spp. and TBEV in host-seeking ticks were published in 2004 and 2013, respectively [24–29]. Therefore, in the current large-scale countrywide study, the prevalence of TBEV, *Borrelia*, *Babesia*, *Anaplasma* and *Rickettsia* pathogens was studied in all three medically important tick species.

### *Rickettsia* spp.

Tick-borne rickettsiae are considered to be emerging; thus, it is important to obtain comprehensive data on the occurrence and prevalence of spotted fever group rickettsiae across Europe. In this study, *Rickettsia* spp. were detected in nearly 20% of all ticks. Three *Rickettsia* species were identified: *R. helvetica*, *R. monacensis* and *R. raoultii*. *Rickettsia helvetica* has been detected in *Ixodes* ticks in many European and Asian countries, and there is evidence that it may cause disease in humans [30]. On the other hand, cases of *R. monacensis* infection in humans have been reported in Spain, Italy, the Netherlands and South Korea [31]. In our study, both *R. helvetica* and *R. monacensis* were mainly associated with *I. ricinus* ticks, and infection rates were similar to those reported earlier in eastern European countries [32]. Additionally, *R. helvetica* was found in *D. reticulatus* ticks in our study (5.21%), and 17.82% of the specimens of this tick species were *R. raoultii*-positive. *Rickettsia raoultii* is frequently detected in multiple tick species and, along with *R. slovaca*, is a causative agent of a syndrome in humans known as DEBONEL/TIBOLA (*Dermacentor*-borne necrosis erythema and lymphadenopathy/tick-borne lymphadenopathy), a newly recognized emerging disease [33, 34]. Thus, the results of this study confirmed that the spread of novel vectors could bring with it an additional risk of exposure to novel emerging pathogens. Moreover, small proportions of *I. ricinus* and *I. persulcatus* ticks were *R. raoultii-*positive in this study, and some of these samples were collected in Latvian regions where *D. reticulatus* ticks are absent. These findings indicate the involvement of other factors, such as the availability of suitable reservoir animals, in the spread of this emerging pathogen.

### *Anaplasma phagocytophilum*

The tick-borne pathogen *A. phagocytophilum* is of both public health and veterinary importance. This pathogen is a generalist, has a very broad range of hosts and causes disease in many mammalian species, including humans [35]. Human granulocytic anaplasmosis occurs in America, Europe and Asia [36]. The main vector of *A. phagocytophilum* in Europe is *I. ricinus*, but this pathogen has also been found in other tick species, including *D. reticulatus* [37, 38]. The prevalence in questing ticks ranges from 0.4% to 33.9% in some localities, and due to the wide distribution of suitable hosts, *A. phagocytophilum* is found in all European countries [35]. In our study, *A. phagocytophilum* was identified at similar proportions in all three tick species, and its overall prevalence was 1.09%. This finding confirmed the risk to humans and animals.

### *Borrelia* spp.

In Latvian *Ixodes* ticks, *Bo. miyamotoi*, a member of the relapsing fever group spirochaetes, as well as the Lyme-disease spirochaetes *Bo. burgdorferi* (*s.s.*), *Bo. afzelii*, *Bo. garinii*, *Bo. valaisiana* and *Bo. lusitaniae* were recorded, among which *Bo. afzelii* was the predominant species (388/657, 59.1%). The total prevalence of *Bo. burgdorferi* (*s.l.*) spirochaetes in *I. persulcatus* ticks was significantly higher than that in *I. ricinus* (23.42 *vs* 15.99%); this result is in accordance with previous studies conducted in regions of sympatric tick occurrence [39, 40]. Additionally, in a meta-analysis conducted in Europe, the reported prevalence of *Bo. burgdorferi* (*s.l.*) in *I. ricinus* was 18.6% [41]. This is the first report of *Bo. miyamotoi* in field-collected ticks in Latvia. This pathogen was found in both *I. ricinus* and *I. persulcatus* ticks, and the prevalence rates (1.15% and 1.27%, respectively) were in accordance with the results of studies conducted in the neighboring countries of Estonia, Sweden and Finland [39, 42, 43]. Additionally, among the 595 *D. reticulatus* ticks, six (1.01%) were positive for *Bo. burgdorferi* (*s.l.*); both *Bo. afzelii* and *Bo. garinii* were detected. The presence of Lyme disease pathogens in *D. reticulatus* ticks has been previously reported in European countries [18, 44]; thus, it is important to decipher their vector competence for these agents in future studies.

### TBEV

Similar to the situation for Lyme disease pathogens, the vector competence of *D. reticulatus* for TBEV is still unclear. A recent study reported the repeated isolation of TBEV from adult *D. reticulatus* ticks in Germany, and it was suggested that *D. reticulatus* plays an equal role to *I. ricinus* in TBEV circulation when the two tick species are sympatric [45]. In our study, TBEV RNA was detected in three males and one female *D. reticulatus* tick, corresponding to an overall prevalence of 0.67% (4/595). Similar proportions of *I. ricinus* and *I. persulcatus* ticks (i.e. 0.65% and 0.63%, respectively) were TBEV-positive. Previously, it was shown that from 1993 to 2002, the annual TBEV infection rate of field-collected adult ticks among *I. ricinus* adults varied between 1.7% and 26.6%, while for *I. persulcatus*, it varied between 0% and 37.3% [25]. Another study reported that in Latvia, the difference in TBEV prevalence between the two *Ixodes* species was not statistically significant (1.02% for *I. persulcatus* and 1.51% for *I. ricinus*) [28]. In the present study, the three tick species were infected with TBEV at equal rates. Thus, it can be suggested that all three tick species are currently involved in the circulation of TBEV in nature in sympatric regions. Additionally, the results of our study revealed the existence of TBEV-positive samples mainly in localities in the western Kurzeme region. This finding is in accordance with epidemiological data showing that the highest incidence of TBE in Latvian patients is observed in the same region (Kurzeme, 19.3 cases per 100,000) [46]. This finding highlights the importance of the surveillance of vector-borne pathogens in nature.

### *Babesia* spp.

Within *Babesia*, *Ba. microti*, *Ba. venatorum*, *Ba. capreoli* and *Ba. canis* were detected in this study. Both *Ba. microti* and *Ba. venatorum* are considered to pose a zoonotic risk to humans [47]. In total, 21 *I. ricinus* ticks (0.55%) carried *Ba. microti*, and 41 (1.07%) carried *Ba. venatorum*. Additionally, a single *I. persulcatus* tick was *Ba. microti*-positive in this study (0.63%). Overall, these findings are similar to the results of our previous study in *Ixodes* ticks collected during 2005–2007, except that both *Ba. microti* and *Ba. venatorum* were detected in *I. persulcatus* ticks in the previous study [26]. The geolocation data indicated that both *Babesia* species were present in different regions of Latvia. On the other hand, 0.91% of *I. ricinus* ticks (35/3840) carried *Ba. canis*, the agent of canine babesiosis, which is usually strongly associated with *D. reticulatus* ticks. The presence of *Ba. canis* in *I. ricinus* observed here is in accordance with a study conducted by Cieniuch et al. [48] in Poland, which showed that approximately 1% of field-collected *I. ricinus* ticks were infected, and also agrees with our recent study on ticks collected from dogs [10]. Interestingly, in the present study, the prevalence of *Ba. canis* in field-collected *D. reticulatus* was very low, as only two samples out of 595 (0.34%) were positive for this species. In contrast, 14.8% of *D. reticulatus* ticks removed from Latvian dogs were *Ba. canis-*positive [10]. In a study by Radzijevskaja et al. [27], the overall prevalence of *Babesia* in *D. reticulatus* ticks from southern Latvia was 2.8%; however, the prevalence of different species was not studied. Based on the molecular screening of field-collected ticks, the prevalence of *Ba. canis* in adult *D. reticulatus* ticks in Europe varies from 0% to 14.8% (see [6] for a review). In a recent study, *Ba. canis* was present in 0.9% of *D. reticulatus* ticks in north-western Europe [49]. Additionally, our analysis of the geolocation data for *Ba. canis*-positive samples (disregarding the tick species) revealed the presence of several compact, geographically separated foci, suggesting an uneven, mosaic-like distribution in nature. Similarly, in a recent study in England, most of *D. reticulatus* ticks at a collection site related to an outbreak of canine babesiosis were *Ba. canis-*positive, while in other locations, all but one of the ticks were *Ba. canis* negative [50]. The existence of endemic foci of infected vectors indicates the existence of favorable conditions for the spread of parasitic diseases and their vectors.

### Prevalence of co-infections

Importantly, the presence of two or three pathogens was found in a considerable portion of host-seeking ticks in our study. The total co-infection rate was 5.55%; however, co-infections were found significantly more often in *I. ricinus* (6.3%) than in *D. reticulatus* (1.34%) ticks; 3.16% of *I. persulcatus* ticks were also positive for more than one pathogen. High variability of co-infections was observed. The coexistence of multiple pathogens in *Ixodes* ticks has been reported in the USA, Europe and Asia, and most co-infections involve two of the three major human pathogens, i.e. *Bo. burgdorferi* (*s.l.*), *A. phagocytophilum* and *Babesia* spp. (see [23] for a review). In Europe, co-infections are detected in up to 13% of *Ixodes* ticks [51]. Thus, the possibility of co-infection with more than one tick-borne pathogen exists for both humans and animals, particularly in endemic areas. Co-infection generally increases the diversity of presenting symptoms, and the disease course may be prolonged and more severe [52]. In our study, the co-infection of *Bo. burgdorferi* (*s.l.*) with *Rickettsia* was observed most frequently; however, the total number of different pathogen combinations was as high as 57. Thus, awareness regarding possible co-infections in ticks should be increased, and further studies are needed, especially in the background of climate change and the emergence and spread of sympatric areas for *Ixodes* and *Dermacentor* tick species.

## Conclusions

This countrywide, large-scale study provides a snapshot of the current distribution patterns of *Ixodes* and *Dermacentor* ticks in Latvia and gives us a reliable overview of tick-borne pathogens in field-collected ticks. The ongoing changes in tick abundance and distribution patterns along with changes in tick-borne pathogen diversity and prevalence indicate the importance of surveillance and future epidemiological studies.

## Supplementary information

**Additional file 1: Table S1.** Prevalence of co-infections in field-collected ticks in Latvia, 2017–2019 (*n* = 4593).

## Data Availability

All data generated or analyzed during this study are included in this published article.

## Abbreviations

95% CI: 95% confidence interval
EDTA: ethylenediaminetetraacetic acid
OR: odds ratio
PCR: polymerase chain reaction
*s.l.*: *sensu lato*
*s.s.*: *sensu stricto*
TBE: tick-borne encephalitis
TBEV: tick-borne encephalitis virus
