# Supplementary material for: Large-scale countrywide screening for tick-borne pathogens in field-collected ticks in Latvia during 2017–2019
Source: Parasit Vectors. 2020 Jul 14;13:351. doi: 10.1186/s13071-020-04219-7 (PMC7362577; doi:10.1186/s13071-020-04219-7)
Supplement: Supplementary file 1 — Additional file 1: Table S1. Prevalence of co-infections in field-collected ticks in Latvia, 2017–2019 (n = 4593). [file 13071_2020_4219_MOESM1_ESM.pdf]

Additional file 1: Table 1. Prevalence of co-infections in field-collected ticks in Latvia, 2017-2019 (*n* = 4593).

	I. ricinus								***I. persulcatus***								***D. reticulatus***								**All ticks**	
	Male		Female		Nymph		Total		Male		Female		Nymph		Total		Male		Female		Nymph		Total		**Total**	
**Co-infections**	No.	%	No.	%	No.	%	No.	%	No.	%	No.	%	No.	%	No.	%	No.	%	No.	%	No.	%	No.	%	**No.**	**%**
A. phagocytophilum + *Ba. canis*			1	0.08			1	0.03																	**1**	**0.02**
Bo. afzelii + *A. phagocytophilum*	1	0.07	1	0.08			2	0.05																	**2**	**0.04**
Bo. afzelii + *Ba. canis*	5	0.37	3	0.23			8	0.21																	**8**	**0.17**
Bo. afzelii + *Ba. microti*	3	0.22	6	0.45	1	0.09	10	0.26			1	1.35			1	0.63									**11**	**0.24**
Bo. afzelii + *Bo. miyamotoi*			1	0.08			1	0.03																	**1**	**0.02**
Bo. afzelii + *Ba. venatorum*	2	0.15	2	0.15			4	0.10																	**4**	**0.09**
Bo. afzelii + *R. helvetica*	26	1.91	33	2.49	4	0.35	63	1.64					1	14.29	1	0.63			1	0.24			1	0.17	**65**	**1.42**
Bo. afzelii + *Bo. miyamotoi* + *R. helvetica*	1	0.07					1	0.03																	**1**	**0.02**
Bo. afzelii + *Bo. burgdorferi* (*s.s.*) + *Ba. canis*					1	0.09	1	0.03																	**1**	**0.02**
Bo. afzelii + *R. helvetica* + *Ba. canis*			1	0.08			1	0.03																	**1**	**0.02**
Bo. afzelii + *R. helvetica* + *Ba. microti*	1	0.07	3	0.23			4	0.10																	**4**	**0.09**
Bo. afzelii + *R. helvetica* + *Ba. venatorum*					1	0.09	1	0.03																	**1**	**0.02**
Bo. afzelii + *R. raoultii* + *A. phagocytophilum*	1	0.07					1	0.03																	**1**	**0.02**
Bo. afzelii + *R. raoultii*	3	0.22	4	0.30			7	0.18																	**7**	**0.15**
Bo. afzelii + *Ba. microti*			1	0.08			1	0.03																	**1**	**0.02**
Bo. afzelii + *Bo. garinii*											1	1.35			1	0.63									**1**	**0.02**
Bo. burgdorferi (*s.s.*) + *R. helvetica*	4	0.29	2	0.15			6	0.16																	**6**	**0.13**
Bo. garinii + *Bo. burgdorferi* (*s.s.*)	1	0.07					1	0.03																	**1**	**0.02**
Bo. burgdorferi (*s.s.*) + *R. helvetica* + *R. raoultii*	1	0.07					1	0.03																	**1**	**0.02**
Bo. garinii + *Bo. valaisiana*			1	0.08			1	0.03																	**1**	**0.02**
Bo. garinii + *Ba. venatorum*	2	0.15					2	0.05																	**2**	**0.04**
Bo. garinii + *Bo. valaisiana* + *R. helvetica*			1	0.08			1	0.03																	**1**	**0.02**
Bo. garinii + *Bo. lusitaniae* + *R. helvetica*	1	0.07					1	0.03																	**1**	**0.02**
Bo. garinii + *R. helvetica* + *Ba. venatorum*					1	0.09	1	0.03																	**1**	**0.02**
Bo. garinii + *R. helvetica*	3	0.22	7	0.53	4	0.35	14	0.36			1	1.35			1	0.63			1	0.24			1	0.17	**16**	**0.35**
Bo. garinii + *R. raoultii*			3	0.23			3	0.08																	**3**	**0.07**
Bo. garinii + *R. raoultii* + *A. phagocytophilum*			1	0.08			1	0.03																	**1**	**0.02**
Bo. garinii + *Bo. lusitaniae*			3	0.23			3	0.08																	**3**	**0.07**
Bo. lusitaniae + *A. phagocytophilum*	1	0.07					1	0.03																	**1**	**0.02**
Bo. lusitaniae + *R. monacensis*	1	0.07					1	0.03																	**1**	**0.02**
Bo. lusitaniae + *R. helvetica*	13	0.95	8	0.60	1	0.09	22	0.57																	**22**	**0.48**
Bo. lusitaniae + *R. raoultii*			4	0.30			4	0.10																	**4**	**0.09**
Bo. lusitaniae + *Bo. valaisiana*	2	0.15					2	0.05																	**2**	**0.04**
Bo. miyamotoi + *A. phagocytophilum*			1	0.08			1	0.03																	**1**	**0.02**
Bo. miyamotoi + *Ba. venatorum*	1	0.07					1	0.03																	**1**	**0.02**
Bo. miyamotoi + *R. helvetica*	3	0.22	2	0.15	1	0.09	6	0.16																	**6**	**0.13**
Bo. miyamotoi + *R. helvetica* + *Ba. venatorum*			1	0.08			1	0.03																	**1**	**0.02**
Bo. miyamotoi + *R. raoultii*			1	0.08			1	0.03																	**1**	**0.02**
Bo. valaisiana + *R. helvetica*	4	0.29	6	0.45			10	0.26																	**10**	**0.22**
Bo. valaisiana + *R. raoultii*			1	0.08			1	0.03																	**1**	**0.02**
R. helvetica + *A. phagocytophilum*	7	0.51	1	0.08	1	0.09	9	0.23																	**9**	**0.20**
R. helvetica + *Ba. canis*	2	0.15	1	0.08			3	0.08											1	0.24			1	0.17	**4**	**0.09**
R. helvetica + *Ba. microti*			1	0.08			1	0.03																	**1**	**0.02**
R. helvetica + *Ba. venatorum*	4	0.29	1	0.08			5	0.13																	**5**	**0.11**
R. helvetica + *R. raoultii*	8	0.59	3	0.23	5	0.43	16	0.42									1	0.58	1	0.24			2	0.34	**18**	**0.39**
R. helvetica + *R. raoultii* + *Ba. venatorum*			1	0.08	1	0.09	2	0.05																	**2**	**0.04**
R. helvetica + *R. monacensis*			1	0.08			1	0.03																	**1**	**0.02**
R. raoultii + *A. phagocytophilum*			1	0.08			1	0.03																	**1**	**0.02**
R. raoultii + *Ba. canis*																			1	0.24					**1**	**0.02**
R. raoultii + *Ba. venatorum* + *Ba. capreoli*			1	0.08			1	0.03																	**1**	**0.02**
TBEV + *Bo. afzelii*	1	0.07	1	0.08			2	0.05	1	1.30					1	0.63									**3**	**0.07**
TBEV + *Bo. afzelii* + *R. helvetica*	1	0.07	1	0.08			2	0.05																	**2**	**0.04**
TBEV + *Bo. burgdorferi* (*s.s.*)	1	0.07					1	0.03																	**1**	**0.02**
TBEV + *Bo. garinii*	1	0.07					1	0.03																	**1**	**0.02**
TBEV + *Bo. valaisiana* + *R. raoultii*			1	0.08			1	0.03																	**1**	**0.02**
TBEV + *R. helvetica*	2	0.15	1	0.08	1	0.09	4	0.10																	**4**	**0.09**
TBEV + *R. raoultii*																	2	1.16					2	0.34	**2**	**0.04**
Co-infections total	107	7.85	113	8.53	22	1.91	242	6.30	1	1.30	3	4.05	1	14.29	5	3.16	3	1.74	5	1.19			8	1.34	**255**	**5.55**
Total number of ticks	1363		1324		1153		3840		77		74		7		158		172		419		4		595		**4593**	
